# Fluence-map generation for prostate intensity-modulated radiotherapy planning using a deep-neural-network

**DOI:** 10.1038/s41598-019-52262-x

**Published:** 2019-10-30

**Authors:** Hoyeon Lee, Hojin Kim, Jungwon Kwak, Young Seok Kim, Sang Wook Lee, Seungryong Cho, Byungchul Cho

**Affiliations:** 1 Department of Nuclear and Quantum Engineering, Korea Advanced Institute of Science and Technology, Daejeon, South Korea; 2 Department of Radiation Oncology, Asan Medical Center, University of Ulsan College of Medicine, Seoul, South Korea

**Keywords:** Prostate cancer, Radiotherapy

## Abstract

A deep-neural-network (DNN) was successfully used to predict clinically-acceptable dose distributions from organ contours for intensity-modulated radiotherapy (IMRT). To provide the next step in the DNN-based plan automation, we propose a DNN that directly generates beam fluence maps from the organ contours and volumetric dose distributions, without inverse planning. We collected 240 prostate IMRT plans and used to train a DNN using organ contours and dose distributions. After training was done, we made 45 synthetic plans (SPs) using the generated fluence-maps and compared them with clinical plans (CP) using various plan quality metrics including homogeneity and conformity indices for the target and dose constraints for organs at risk, including rectum, bladder, and bowel. The network was able to generate fluence maps with small errors. The qualities of the SPs were comparable to the corresponding CPs. The homogeneity index of the target was slightly worse in the SPs, but there was no difference in conformity index of the target, V_60Gy_ of rectum, the V_60Gy_ of bladder and the V_45Gy_ of bowel. The time taken for generating fluence maps and qualities of SPs demonstrated the proposed method will improve efficiency of the treatment planning and help maintain the quality of plans.

## Introduction

Radiotherapy is an essential in cancer treatment and is used for approximately 50% of all cancer patients^1^. The radiotherapy works by delivering high-energy radiation to the tumour. Since the radiation cannot distinguish cancer cells from normal tissues, it is important to create plans that can deliver a high dose to cancer cells while minimising the dose to normal cells. One of the methods to accomplish the goal is using intensity modulation in the delivery. This method utilises a multi-leaf collimator (MLC) located between the patient and radiation source to modulate and shape the high energy photon beam^2^. The shape and delivery intensity of the photon beam is determined by an optimisation process called inverse planning. Therefore, it is important to create high quality plans. However, differences in planning skills between planners can influence plan quality^3^, which means that patients may undergo different quality of treatments. Many studies have attempted to automate treatment planning to maintain plan quality, including those that have predicted dose-volume-histograms (DVH) for treatments^4–6^. The DVH is a common criterion for the inverse planning procedure; however, it does not provide spatial information of the dose^7^. In automated treatment planning, optimisation based on dose volume objectives on DVH may lead to unwanted plans. To improve the quality of automated plans, some studies have predicted the spatial dose distribution from patient images or contour data^7–9^. Those studies predicting DVHs^4^ or spatial dose distributions^9^ by using hand-crafted features from statistical analysis, such as the distance from the target or organs-at-risk (OARs), and the volume of the target and learning-based methods trained by the hand-crafted features.

Recently, deep-neural-networks (DNNs) have shown outstanding results in various fields including radiotherapy^10–12^, and there are studies using DNNs to predict the dose distribution of treatments^13,14^. Deep learning-based dose prediction methods do not require the hand-crafted features used in previous studies. A network typically consists of convolution, pooling and fully-connected layers with activation functions, and takes patient images or contour data as input data. In the training phase, the layers and activation functions learn high dimensional features that can predict dose distributions from given input data.

In previous studies, the predicted dose distributions or DVHs were used to identify suboptimal plans or to guide the plan optimisation process, to reduce time consumption and help planners to improve their plans and maintain quality between different planners. Some studies have generated deliverable plans from predicted dose distributions using dose mimicking^15^ or fluence-map optimisation methods constrained by the spatial dose distribution^16^. These studies utilised methods that can perform optimisation based on voxel dose constraints. This requires an optimisation step to determine the delivery parameters, which is one of the most time-consuming steps in the creation of treatment plans, as it is performed in an iterative manner to figure out the optimal solution meeting the criteria. Furthermore, different parameters for the optimisation algorithm influence the quality of plans.

Following the success of previous studies predicting dose-maps from contour data, we here focus on generating optimal fluence maps directly from organ contours and the dose-map. We assumed that dose-maps obtained from previous studies were clinically acceptable, and used a DNN to generate fluence maps from organ contours and dose distributions without an optimisation step. Direct generation of fluence maps can reduce the time and effort required in performing optimisation. Moreover, it will help to reduce variation between planners and therefore help to maintain the quality of treatment plans.

## Materials and Methods

### Proposed method

**Figure 1:**
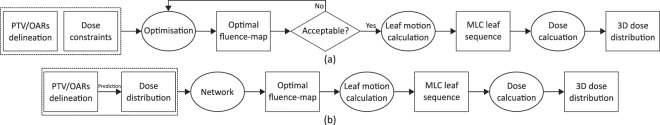
(**a**) The conventional IMRT inverse planning process and (**b**) the proposed framework based on a deep-neural-network.

In conventional IMRT planning, the planning target volume (PTV) and OARs are determined on the planning CT images and the dose is prescribed. Using the prescription and organ contours, optimisation algorithms determine the optimal fluence maps to meet the optimisation objectives for the PTV and OARs, which are imposed on the DVH, as summarised in Fig. 1(a). In Fig. 1, the items inside circles are processes and the items inside the rectangles are products. In the optimisation step, various parameters are tuned to achieve the optimal solution and this process is continued until an acceptable fluence map is achieved.

In this study, we propose a framework to generate fluence maps directly from the organ contours and dose distributions. The proposed framework for deep-learning based IMRT planning is summarised in Fig. 1(b). As shown in the figure, we trained a neural network to directly find the optimal fluence maps of beams from organ contours and dose distributions.

By generating fluence maps, we can create deliverable treatment plans without a parameter tuning process and faster than can be done using optimisation algorithms; therefore, we can increase the efficiency of the planning process. As noted in the introduction, we assumed that volumetric dose distributions with clinically acceptable qualities were obtained from the previous studies and regarded the clinical dose distributions as prediction results.

### Training data collection

For training purposes, we collected data from 285 prostate cancer patients under the approval of the Institutional Review Board (IRB) of Asan Medical Center for a retrospective study in accordance with guidelines for protecting patients’ private information. The informed consent was waived based on the nature of a retrospective study. There are two phases in the training process of a DNN: training and validation phases. In the training phase, the weights in the DNN are updated based on the input and its associated desirable output by use of the training dataset. The validation phase checks generality of the trained weights using separate and unused set of input and desirable output data from the dataset. After the training is done, we provide test input data to the network for evaluation. We compared the output from the network to the clinical data for evaluating the performance of the network. Among the 285 patients’ data, 240 patients’ data were used for the training and validation, and the remaining 45 patients’ data were used for the evaluation. The data were randomly selected in the entire study.

**Figure 2:**
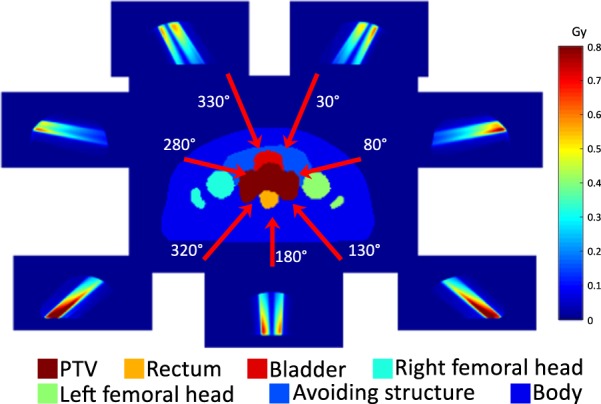
Organ contours, directions of treatment beams and dose distributions per beam.

Patients were instructed to empty their bowels and bladder immediately before the planning CT scan. An endorectal balloon was used to improve the position of the rectum, and after insertion and inflation with 60 cc of air, the balloon was fixed in place with an individual adjustable stopper. Details on the planning CT acquisitions have been described elsewhere^17,18^. All of the patients underwent 15 megavolt (MV) 7-field IMRT. For most of the plans, the radiotherapy intention was to treat the tumour or tumour bed and the regional pelvic nodes. Based on the institutional protocol, the patients underwent the initial treatment of 44 Gy in 20 fractions to the tumour or tumour bed including the regional pelvic nodes, and were followed by the boost treatment of 28.6 Gy in 13 fractions to the prostate/bed. In this study, we only used the initial treatment plans. Treatments were performed on Clinac, TrueBeam, or VitalBeam machines (Varian Medical Systems, Inc., Palo Alto, CA, USA). The beam directions and corresponding dose distributions for a typical case are shown in Fig. 2. The dose distributions are presented in Gy and their values are indicated by the colour bar on the right of the figure.

For the organ contour data, we labelled ten organs/structures in the patients with ten different integer values for use in training. These ten labels were ‘Body’, ‘Virtual avoiding structures’, ‘Bowel’, ‘Right femoral head’, ‘Left femoral head’, ‘Penile bulb’, ‘Rectum’, ‘Prostate’, ‘Bladder’ and ‘PTV’. Virtual avoiding structures are structures used to add detailed dose constraints to OARs, like bladder, bowel or rectum. Two virtual avoiding structures were generated for inverse planning purposes; one included the intestinal and pelvic cavity area that was not specified by bowel, bladder or rectum, and the other one included the regions posterior to the rectum. We labelled the same values for the both virtual avoiding structures.

We normalised the dose distributions to a single fraction to utilise the dose distributions for training.

### Training of the DNN

**Figure 3:**
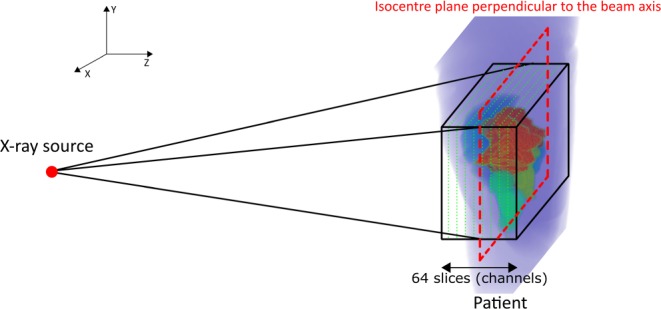
Geometry of the contour resampling.

**Figure 4:**
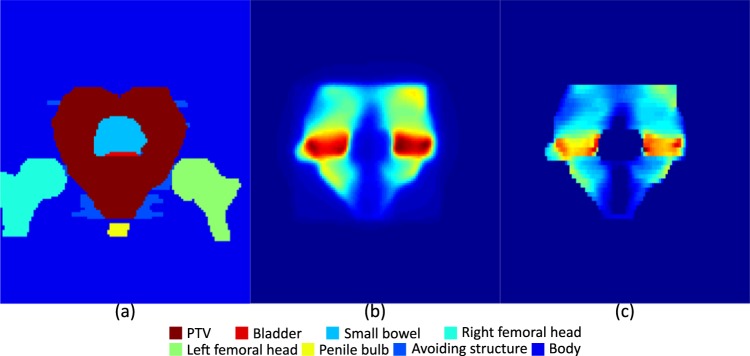
Images from the 32^nd^ channel of input (on the isocentre plane perpendicular to the beam axis) and output data for a beam at 180°. (**a**) Organ contours, (**b**) dose distribution for the beam direction and (**c**) corresponding fluence-map.

For the training, we used organ contours, containing PTV and OARs, and dose distributions, viewed from the beam’s eye view (BEV) of a single beam, as input data and used fluence map at each corresponding beam direction as desired output data. As every treatment had seven fields, we had seven different input data for each individual patient. We matched the dimensions of organ contours and dose distributions to those of the fluence maps by resampling the data to the dimensions of the fluence maps. We also divided the input data into 64 different slices according to the distance from the beam source and considered the cone characteristics of the source, as shown in Fig. 3 and each slice was considered as different channel of input data. In Fig. 3, the target and OARs are located inside the black rectangle, the plane surrounded by the red dashed-lines depicts a plane located on the central axis and normal to the photon source, and the rectangles surrounded by green dashed-lines indicate slices used for resampling. After resampling, the input data was the same size as the fluence maps, with 64 channels on the direction of beams, each having a 10 mm thickness. We divided the contours into channels to provide 3D positional information to the network. Because of the attenuation characteristics of the photon beam, the dose decreases as the beam penetrates. Therefore, a beamlet incident on any OAR prior to the PTV would be less encouraged compared with a beamlet firstly incident on the PTV. Increasing the number of slices increases the resolution of the data; however, it also requires more memory for training because of the increased size of the input data. Some organs may overlap in a single channel because of the coarse pixel size of the channels; in such cases, we preserved the organs with a higher label number. In Fig. 4(a,b), the images of the organ contour data and dose distributions for the 32^nd^ channel of a treatment beam at 180° are presented as an example of the input data with corresponding desired fluence maps in Fig. 4(c).

As mentioned above, we considered each treatment field as an individual data. Although this gave us a total of 1680 (7 × 240) data for training and validation, this is still rather a small amount of data for training a DNN. Therefore, we augmented the data by translating the data in 80 different sets of directions, with each set having different values for x- and y-shift values. We only used translation for augmentation to avoid pixel value changes due to interpolation while rotating the data and excluded data if truncation occurred in dose distribution or PTV contours after translation. After the augmentation, we had 128,666 data. The numbers of training and validation datasets after the augmentation are shown in Table 1.

**Table 1:** Parameters for training the network.

Parameters	Values
Number of training datasets	115800
Number of validation datasets	12866
Optimizer	Adam optimizer
Base learning rate	0.001
Batch size	15
Number of training epoch	100 epochs

**Figure 5:**
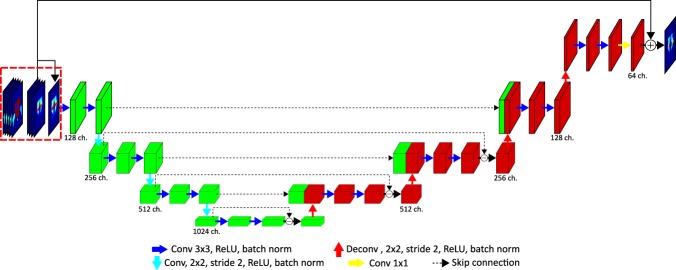
Structure of the network used for training.

The network used for training is shown in Fig. 5. We modified the structure of the dual frame U-Net^19^ to suit our purpose. This network is an alternative implementation of the tight frame U-Net, which showed better performance in preserving high frequency components while training. We used the structural similarity (SSIM)^20^ index and the mean-absolute error with equal weighting factors as cost functions. To use the SSIM for training, we used a convolution operation with a Gaussian kernel to calculate the mean and standard deviation^21^. For faster convergence of the network, we used a residual learning scheme. We gave the central slice of the dose as an initial guess, and then trained the network to learn the difference between the initial guess and the ground truth data. In the training phase, three different input data were provided to the network: 3D organ contours, the corresponding 3D dose distribution and the central channel of the dose distribution, as shown in Fig. 5 (at the beginning of the network and outlined with a red box). At the end of the network, the central channel of the dose distributions, the third input data, was added to the output of the network for residual learning and the summation result was compared with the fluence maps to update the kernels. We used strided-convolution with 2*2 sized kernels and stride 2 for down-sampling and transposed convolution (also called deconvolution) for up-sampling with the same kernel size and stride used for the strided-convolution. For the last transposed-convolution, the size of the data was not changed, while the number of channels was reduced by half.

Details of the training parameters are summarised in Table 1. While training, we reduced the learning rate by 0.8 every 10 epochs to increase the convergence of the network. In addition, we used a gradient clipping method^22^ to avoid a gradient exploding problem. This method calculates the norm of the gradient of each kernel and multiplies the ratio of the calculated norm and predefined value to the gradients if the calculated norm is bigger than a predefined value. We set the predefined value to $1{{\rm{e}}}^{-4}$. If the norm of the gradients of a kernel was larger than the value, the gradients were scaled down to make their norm the same as the value. If the norm was smaller than the value, normalisation was not performed and the original gradients were used for the update. For the update, we used an Adam optimizer^23^.

We trained the network on a workstation with an Intel Xeon E5 2.4 GHz processor, 256 GB of RAM and a GTX 1080 Ti with 11 GB of GPU memory.

## Results

### Training results

**Figure 6:**
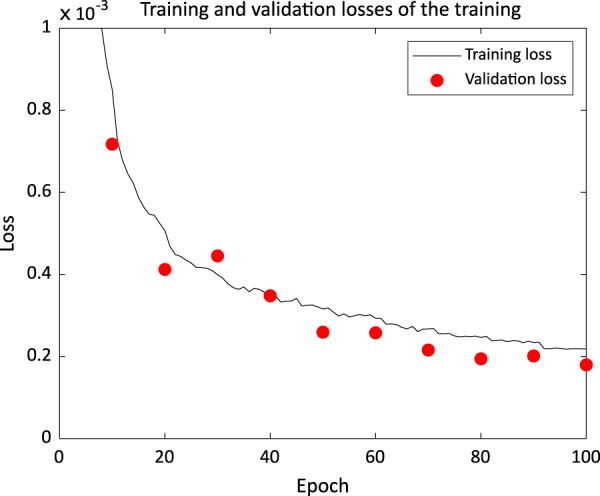
Training and validation losses in training of the network.

We performed validation every ten epochs and the results are shown in Fig. 6 with the training loss. The validation loss curve was acceptably agreeing with the training loss curve as the iteration increases.

To evaluate the performance of the network, we used the network parameters after the last update to generate the fluence maps. We compared the generated fluence maps and the dose distribution calculated from the fluence map to the clinical fluence maps and the clinical dose distribution, respectively.

### Fluence map generation results

**Figure 7:**
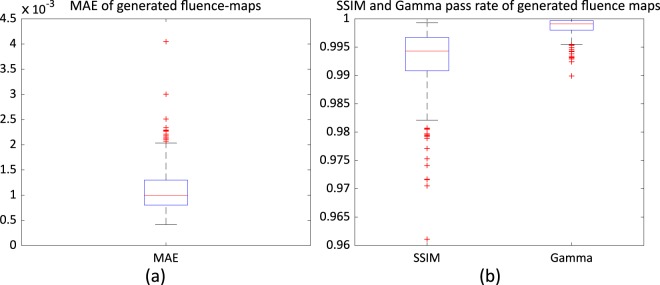
Quantitative comparison results between the generated and clinical fluence maps: (**a**) mean-absolute-error (MAE), and (**b**) structural similarity (SSIM) and gamma pass rate.

As explained in Training data collection section, we had 45 patients’ data reserved for evaluation. We provided organ contours and dose distributions to the network and obtained fluence maps from the network. We calculated the mean-absolute error (MAE) and structural similarity index (SSIM), and performed gamma evaluation with 3 mm/3% criteria between the generated fluence maps and clinical fluence maps. The results are summarised in Fig. 7. The median values of the MAE, SSIM and gamma pass rate were $9.95\times {10}^{-4}$, 0.994 and 0.999, respectively.

**Figure 8:**
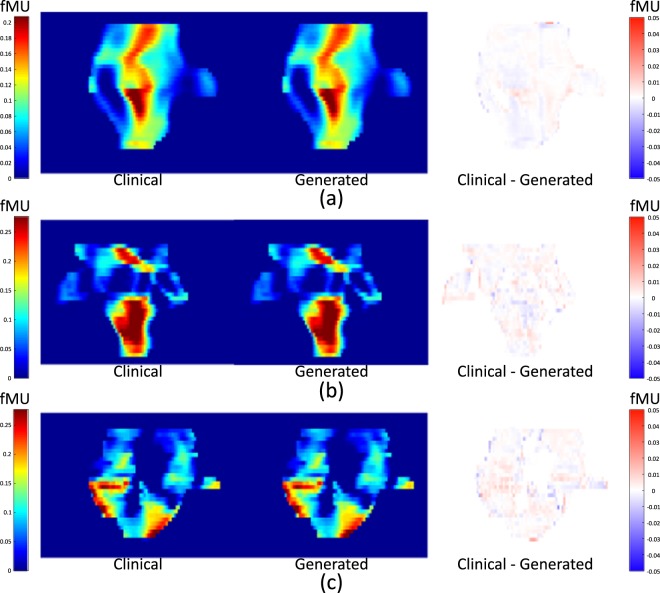
Comparison of the clinical fluence maps with generated fluence maps using the trained network. (**a**) A 230° field for patient #35, (**b**) a 130° field for patient #13 and (**c**) a 330° field for patient #22. fMU stands for fractional MU, which is the delivered MU for a pixel in a fluence map divided by the total MU delivered by a single beam.

Examples of the generated fluence maps are shown in Fig. 8, alongside corresponding clinical fluence maps and their differences. The pixel values of the fluence maps and the differences are presented in fractional monitor units (fMU).

### Synthetic dose distribution results

We calculated dose distributions from the generated fluence maps by feeding them into an Eclipse treatment planning system (Varian Medical Systems, Inc., Palo Alto, CA, USA) as optimal fluence maps, and calculated leaf motion. We calculated volumetric dose distributions of patients using the same treatment planning system based on the calculated leaf sequence. After the dose distributions were calculated, we normalised the plans to have the same mean PTV dose as the clinical plans. In the rest of this paper, we refer to the plans from the generated fluence maps as ‘synthetic plans’ and the dose distributions from these plans as ‘synthetic dose distributions’. We compared the synthetic dose distributions and clinical dose distributions according to the constraints used in our institution^24^, which are homogeneity index (HI), conformity index (CI), V_72.6Gy_ of the PTV, V_60Gy_ of rectum, V_60Gy_ of bladder, D_max_ of femoral heads, D_mean_ of penile bulb and absolute volume of bowel receiving 45 Gy. To calculate HI^25^ and CI^26^, we used Eqs (1) and (2), respectively. In equation (1), D_95%_, D_5%_ and D_PX_ are the dose that 95% of the volume of the PTV is receiving, a dose that 5% of the volume of the PTV is receiving and the prescription dose, respectively. In Eq. (2), V_RI_ is the volume of the reference isodose and V_PTV_ is the volume of the PTV. We used 95% of the prescription dose as the reference isodose, so the volume receiving more than 95% of the prescription dose is divided by the volume of the PTV to calculate CI.

1 $$HI=\frac{{D}_{95 \% }-{D}_{5 \% }}{{D}_{PX}}\times 100$$

2 $$CI=\frac{{V}_{RI}}{{V}_{PTV}}$$

We would like to point out that the criteria were designed for summation of 44 Gy/20 fx of initial treatment including the pelvic nodes and 28.6 Gy/13 fx of boost treatments for the prostate/bed. Since we only considered the initial treatment in this study, we scaled up the initial 44 Gy dose distribution to the total 72.6 Gy to apply the constraints.

**Figure 9:**
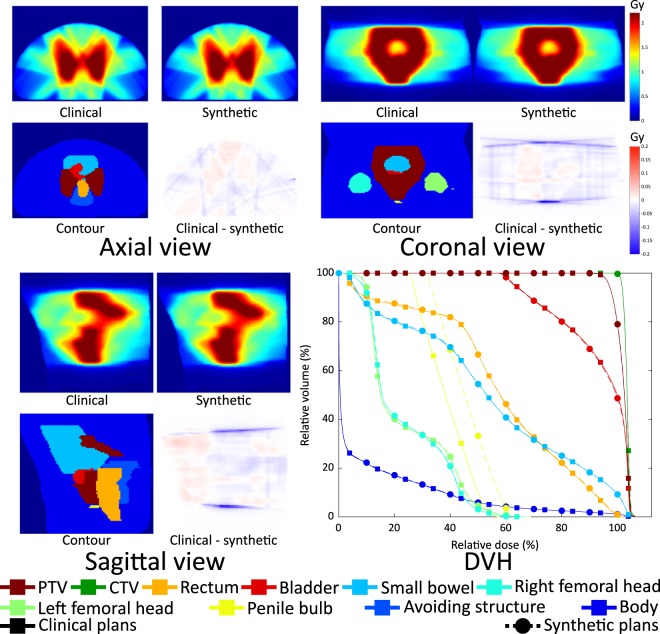
Clinical and synthesised dose distributions, their differences, corresponding contours and DVHs of patient #33 which has the smallest average MAE in the fluence maps.

**Figure 10:**
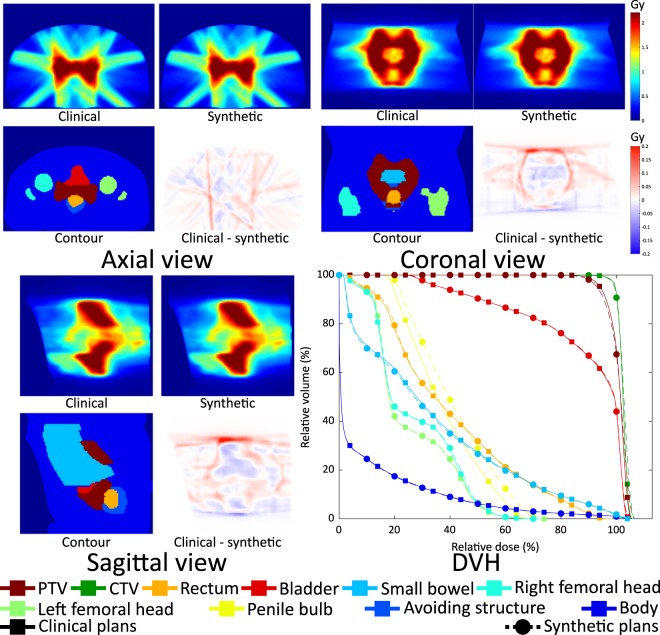
Clinical and synthesised dose distributions, their differences, corresponding contours and DVHs of patient #43 which has the largest average MAE in the fluence maps.

We showed results of two patients in Figs 9 and 10. The dose distributions and DVH curves in Fig. 9 represent the results with the smallest average MAE in the fluence maps. The synthetic dose distributions and its DVH curves show small discrepancies from the clinical ones. In Fig. 10, a result with the largest average MAE in the fluence maps is shown. The larger error in the fluence maps led to a larger error in the dose distributions and a less homogeneous dose being delivered to the PTV.

**Figure 11:**
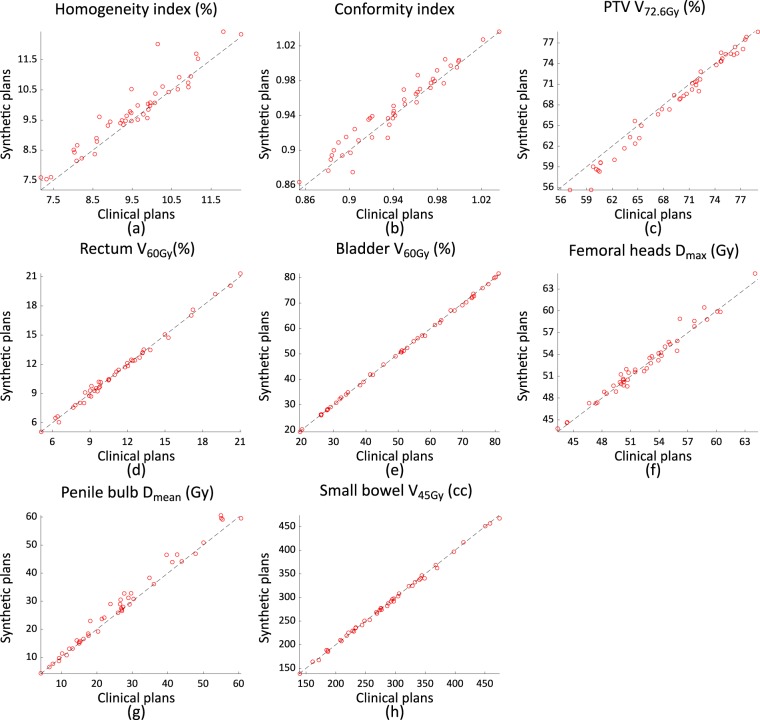
Plan quality comparison results between synthetic plans and clinical plans.

The plan quality metrics are summarised in Fig. 11, with a line indicating identical values. The mean and standard deviation of each criterion of the synthetic and clinical plans are summarised in Table 2, along with the mean and standard deviation of differences of the criteria.

**Table 2:** Means and standard deviations of the quality metrics and their differences.

Metrics	Clinical plans	Synthetic plans	Differences
HI	9.54 (±1.16)	9.78 (±1.18)	−0.24(0.31)
CI	0.94 (±0.04)	0.95 (±0.04)	0.0 (0.01)
PTV V_72.6Gy_ (%)	69.38 (±6.11)	68.52 (±6.66)	0.85 (±0.92)
Rectum V_60Gy_ (%)	11.27 (±3.58)	11.27 (±3.60)	0.00 (±0.26)
Bladder V_60Gy_ (%)	50.76 (±18.90)	50.64 (±18.81)	0.11 (±0.42)
Femoral heads D_max_ (Gy)	52.36 (±4.44)	52.57 (±4.55)	−0.21 (±0.74)
Penile bulb D_mean_ (Gy)	26.60 (±14.68)	28.05 (±15.52)	−1.45 (±2.05)
Small bowel V_45Gy_ (cc)	285.78 (±78.75)	285.01 (±77.84)	0.77 (±3.01)

As shown in Fig. 11 and Table 2, the synthetic plans have comparable quality to clinical plans in terms of saving OARs; however, the dose delivered to the PTV is less homogeneous than that delivered with the clinical plans.

**Figure 12:**
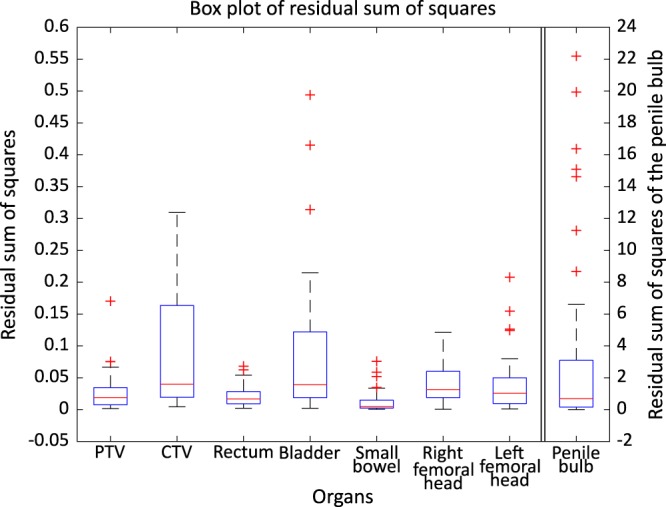
Box plot of RSS of the DVHs of all organs. Left y-axis for PTV, CTV, rectum, bladder, small bowel, right femoral head, and left femoral head and right y-axis for penile bulb.

For a further comparison, we calculated the residual sum of squares (RSS) of the DVHs using Eq. (3) to quantify the discrepancies between the synthetic and clinical plans, and the results are shown in Fig. 12. In Eq. (3), *D* is the relative dose, $DV{H}_{clinical,organ}(D)$ the relative volume of an organ receiving dose *D* in the clinical DVH and $DV{H}_{synthetic,organ}(D)$ is the relative volume of an organ receiving dose *D* in the DVH of synthetic plan.

3 $$RSS(organ)=\mathop{\sum }\limits_{D=0}^{\infty }{(DV{H}_{clinical,organ}(D)-DV{H}_{synthetic,organ}(D))}^{2}$$

The right y-axis of the Fig. 12 indicates RSS value of penile bulb and the left y-axis indicates RSS value of the other organs. We think that large discrepancies in the penile bulb are caused by the small volume of the organ, which makes the DVH for the organ sensitive to small changes in the dose distribution. Apart from the penile bulb DVHs, the other organs show only small discrepancies to the clinical plans.

## Discussion

To our knowledge, this is the first attempt to use a neural network to generate fluence maps from organ contours and dose distributions.

We think major reasons for the errors in the results are that each treatment field is considered as an individual data in the training and that the input data channels have a rather coarse pixel size of 10 mm in this study. By considering each field as an individual data item, the network is not able to take care of influences coming from the other fields, which should be considered in the real world. Training a network with patient-based data (seven treatment fields as an individual data) is expected to increase the performance of the network; however, it simultaneously decreases the number of training datasets, and increases memory requirement for training. Moreover, 3-dimensional positioning of the treatment target makes it difficult to augment the data. Furthermore, some pixels containing multiple organ labels are considered to represent a single organ with higher priority: the PTV in our case, because of the coarse pixel size of the data. If rectum and PTV were to overlap in a channel, all the overlapping pixels would be considered as PTV. Reducing the pixel size of channels or separating different organ contours into different dimensions could help alleviate this problem and should increase the performance of the network; however, this will increase the memory requirements and the time required for training.

As mentioned in the results section, we normalised the plans to have the same mean dose as the clinical plans. In real cases, the reference plan used to adjust the normalisation factor would not exist and physicists should decide on the normalisation factor required to meet the criteria of their institute. We believe that improving the performance of the network will reduce errors of generated fluence maps and lead to the creation of plans without any human interaction.

In order to investigate the contribution of the input data: contour vs. dose distribution, we trained a network using the dose distributions only and compared results with the current network using the contour and dose distributions as input data. The overall performance of both networks was similar. However, fluence maps generated from the network trained with dose distribution only had larger errors of the outliers. Through the comparison, the performance of the network primarily comes from the dose distribution, and the organ contours help to reduce the errors of outliers. One may ask whether labelling order of organ contour affects the network performance. It happened that the PTV had the highest label, OARs close to the PTV had next high, and the tissue away from the target had the smallest label in this study. To check the effect of labelling order, we reversed the order of contour labels and trained the network. The performance of the network trained with a reversed labelling order did not show a noticeable difference from the original network.

The current network took about 10 days to complete 100 epochs, while it takes about a second to generate the fluence maps for each patient. This provides a much faster way than the expert-based manual optimisation methods for fluence map generation. A recent study reported that the manual optimization took 80 minutes on average for the 5-field IMRT plans in 20 prostate cancer patients^27^. Therefore, the proposed method would be particularly helpful for generating adaptive radiotherapy plans and optimising dose delivery for patients while minimising the workload for human practitioners.

We assumed that the dose distributions could be obtained from the aforementioned prediction methods and would be close to the clinical dose distribution in quality. To create a plan for a new patient with our framework, it is important to make a predicted dose distribution in clinical quality. We plan to develop a dose prediction module within the proposed work scope in our future study. Additionally, we are going to build a network that provides the fluence map directly from the organ contour, without dose calculation explicitly, given that a prescribed dose to the target is the same among the patients.

## Conclusion

We propose a framework to generate fluence maps for prostate IMRT using a DNN. The network was trained to generate fluence maps directly from dose distributions and organ contours. The trained network was able to generate fluence maps in a second. This will increase the efficiency of the treatment planning step by shortening the time required to obtain optimal fluence maps and will be helpful to maintain the quality of treatment plans by generating fluence maps directly from organ contours and dose distributions.

## Data Availability

The datasets generated during and/or analysed during the current study are available from the corresponding author on reasonable request.
